# Metaplasticity in the human swallowing system: clinical implications for dysphagia rehabilitation

**DOI:** 10.1007/s10072-021-05654-9

**Published:** 2021-10-16

**Authors:** Ivy Cheng, Shaheen Hamdy

**Affiliations:** Centre for Gastrointestinal Sciences, Division of Diabetes, Endocrinology and Gastroenterology, School of Medical Sciences, Faculty of Biology, Medicine and Health, University of Manchester, Manchester, UK

**Keywords:** Dysphagia, Metaplasticity, Neurostimulation, Neuroplasticity, Rehabilitation, Swallowing

## Abstract

Dysphagia is a common and devastating complication following brain damage. Over the last 2 decades, dysphagia treatments have shifted from compensatory to rehabilitative strategies that facilitate neuroplasticity, which is the reorganization of neural networks that is essential for functional recovery. Moreover, there is growing interest in the application of cortical and peripheral neurostimulation to promote such neuroplasticity. Despite some preliminary positive findings, the variability in responsiveness toward these treatments remains substantial. The purpose of this review is to summarize findings on the effects of neurostimulation in promoting neuroplasticity for dysphagia rehabilitation and highlight the need to develop more effective treatment strategies. We then discuss the role of metaplasticity, a homeostatic mechanism of the brain to regulate plasticity changes, in helping to drive neurorehabilitation. Finally, a hypothesis on how metaplasticity could be applied in dysphagia rehabilitation to enhance treatment outcomes is proposed.

## Introduction

Recovery from brain damage such as stroke is thought to depend, in part, on neuroplasticity, which can be defined as the ability of the nervous system to re-organize its function, structure, and connections in response to intrinsic and extrinsic stimuli [1]. It can occur during development, learning, and in response to brain disruptions or to therapy [1]. Research on rehabilitation of swallowing function after brain injury has started to focus on strategies that promote such plasticity. Neurostimulation techniques, including non-invasive brain stimulation (NIBS) and stimulatory approaches that target the afferent neural pathways of swallowing, have been studied vigorously in recent years and the findings are encouraging [2]. However, there is substantial variability in the responsiveness toward these treatments. Genetic predisposition, brain configuration, and level of neural activation prior to neurostimulation are some of factors that may account for such variability [3]. Among these factors, the neural activation of the motor cortex may be programmed or preconditioned with additional neurostimulation through homeostatic metaplasticity, which refers to the regulation of changes in plasticity [4]. This offers unique opportunities in which the brain could be externally manipulated to achieve less variable treatment outcomes. The present review introduces the mechanisms of neuroplasticity and metaplasticity, summarizes findings on the use of neurostimulation to induce these processes, and discusses the potential application of metaplasticity to improve dysphagia treatment outcomes.

## Neuroplasticity: a key to functional recovery following brain injury

Synaptic plasticity is a form of neuroplasticity. Donald Olding Hebb first proposed that repeated firing of one neuron would result in firing of another functionally connected neuron, leading to an increase in synaptic efficiency [5]. Such synaptic plasticity (Hebbian plasticity) is crucial for establishing new or reinforcing neural connections for functional recovery following stroke [6]. It can occur through long-lasting activity-dependent increases (long-term potentiation; LTP) or decreases (long-term depression; LTD) in synaptic strength [1].

Synaptic (Hebbian) plasticity is regulated by the timely interaction between presynaptic and postsynaptic activity. The direction of plasticity (LTP or LTD) depends on the activation of N-methyl-d-aspartate receptors (NMDARs) and the influx of calcium (Ca^2+^) ions to the postsynaptic neuron [7]. NMDAR activation is maximal when inducing LTP. Following depolarization of presynaptic neuron, glutamate-containing synaptic vesicles bind with the presynaptic membrane and glutamate is released. Glutamate molecules then bind to NMDARs on the postsynaptic membrane, resulting in depolarization of postsynaptic membrane. Sufficient postsynaptic membrane depolarization would result in a release of magnesium (Mg^2+^) ions that block NMDRs and allow influx of Ca^2+^ ions to the postsynaptic cell. This Ca^2+^ ion influx activates the Ca^2+^/calmodulin-dependent kinase II (CaMKII) pathway and a cascade of intercellular responses that alter synaptic efficiency [8]. Conversely, a modest activation of NMDARs and hence a smaller influx of Ca^2+^ ion to the postsynaptic cell is more favorable for the induction of LTD [8].

A number of NIBS techniques, including repetitive transcranial magnetic stimulation (rTMS) and transcranial direct current stimulation (tDCS), have been used to induce long-lasting plasticity changes in the human hand [9, 10] and pharyngeal motor cortex [11, 12]. Such synaptic plasticity changes may result in behavioral or functional changes critical for rehabilitation. Transcranial magnetic stimulation (TMS) is a technique that induces electrical current within the brain through electromagnetic induction. Such electrical currents induced by single pulses of TMS can transiently depolarize the postsynaptic membrane, resulting in action potentials and single-pulse TMS is frequently used as a measure of corticobulbar or corticospinal connectivity [13]. When TMS is applied repetitively (repetitive TMS; rTMS), it can increase or decrease cortical excitability beyond the duration of the stimulation period. For tDCS, direct currents delivered through electrodes on the scalp can penetrate the skull to the brain and modify transmembrane potentials, thereby also changing cortical excitability [10]. In general, high-frequency (> 1 Hz) rTMS and anodal tDCS induce LTP-like plasticity whereas low-frequency (≤ 1 Hz) rTMS and cathodal tDCS induce LTD-like plasticity in the human motor cortex.

Although plasticity plays an important role in brain development and in functional recovery following brain injury, there is a need for checks and balances in the nervous system because synaptic strengthening can be boundless and may result in excitotoxicity by over-excitation of NMDARs and high concentration of neurotoxins that causes cell death [14, 15]. On the other hand, saturation of LTD will lead to synaptic inactivity that compromises the ability of the neural networks to adapt to changes [14, 15]. Therefore, mechanisms to balance and modulate these plasticity changes are necessary.

## Homeostatic metaplasticity: the regulator of plasticity

**Fig. 1:**
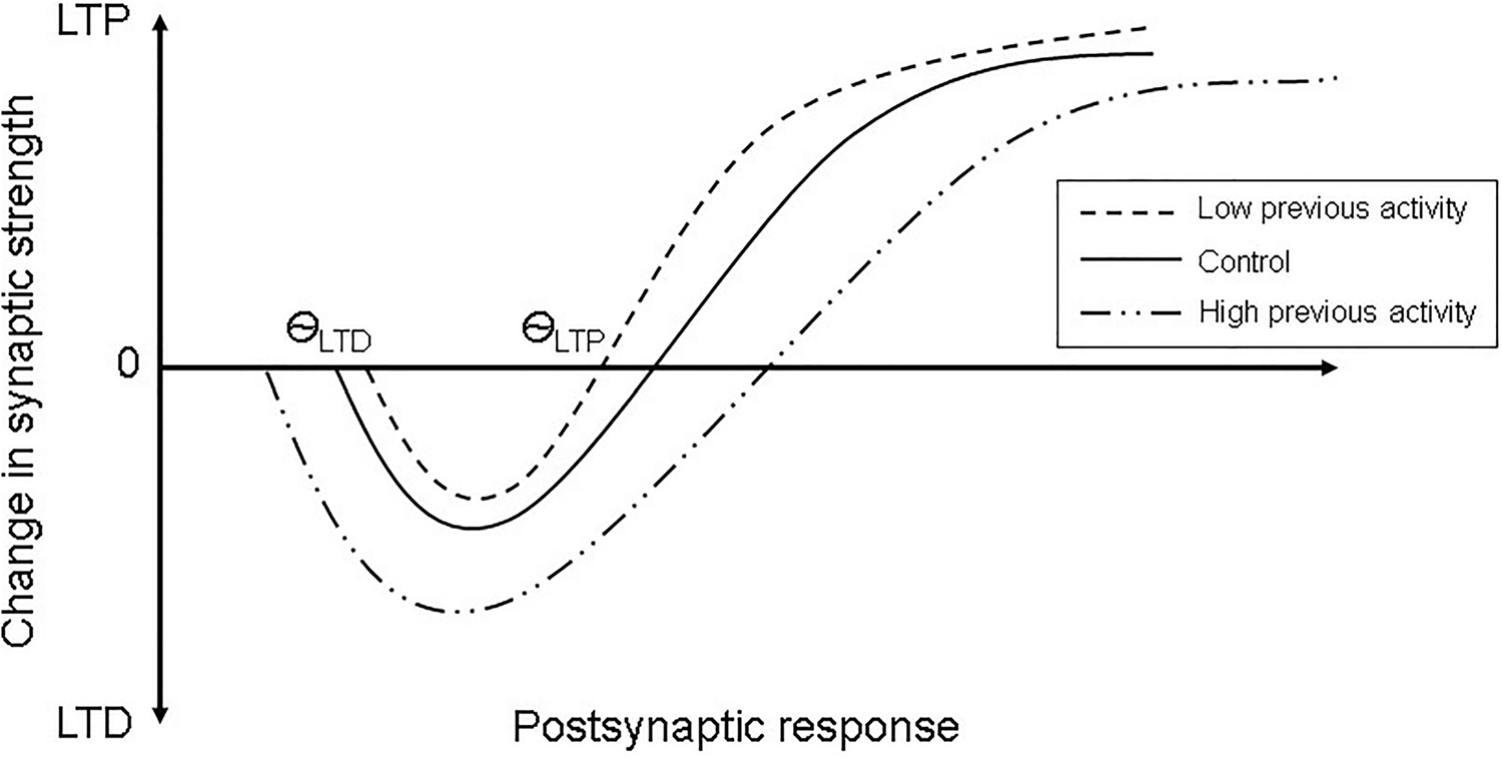
Graphic representation of the Bienenstock-Cooper-Munro (BCM) model. The BCM model states that prior high level of postsynaptic activity lowers the threshold for LTD (*Ѳ*_LTD_) and raises that for LTP (*Ѳ*_LTP_). The converse effect occurs with low previous level of activity

Homeostatic metaplasticity is one such mechanism that regulates plasticity changes and maintains equilibrium at the level of neural activity [4]. This homeostatic mechanism stabilizes the activities of neurons and hence prevents saturation of LTP or LTD within the neural system [15]. Through metaplasticity, the preceding state of synaptic or neural activity can influence the characteristics of the subsequent synaptic changes [15, 16]. Metaplasticity can be explained by the Bienenstock-Cooper-Munro (BCM) computational model [17] (Fig. 1). This model suggested that activity-dependent synaptic activity has a dynamic threshold, which could be modified by preceding postsynaptic activity. The LTD/LTP crossover threshold, termed “modification threshold,” is reduced when there has been preceding low level postsynaptic activity and is increased when there has been preceding high level postsynaptic activity [15–17]. In a study with rat brain slices, Huang et al. [18] demonstrated this phenomenon in the hippocampus. When the synapse was first given either weak tetanic stimulation or single strong shocks, followed by an LTP-inducing stimulation, the LTP induced by the subsequent stimulation were reduced or inhibited. This likely resulted from the activation of NMDARs in response to the first stimuli. The authors postulated that this dynamic influence on the threshold of LTP can prevent a positive feedback loop and hence reduce over-excitation of synapses.

The underlying cellular mechanism for such dynamic influence remains poorly understood, but it is generally accepted that this form of metaplasticity is regulated by the ratio of NMDAR subunits (NR2A/NR2B ratio). NR2A is associated with fast kinetics of excitatory postsynaptic currents whereas NR2B subunit is associated with slow kinetics and strong cellular Ca^2+^ ion entry and LTP [19]. With elevated NR2A/NR2B ratio, the modification threshold is high and stronger stimulation is required to induce LTP whereas with a reduced ratio, weaker stimulation is adequate to induce LTP [7].

### Evidence of metaplasticity from animal and human studies

The BCM model provides the basis for the application of NIBS techniques to induce metaplasticity in human motor cortex. It is hypothesized that preconditioned stimulation can shape the effects of the subsequent stimulation [15]. Iyer et al. [20] first explored metaplasticity properties in the human motor cortex in 25 healthy volunteers. They compared the preconditioning effects of constant frequency (6 Hz), frequency-modulated (ranging from 4 to 8 Hz), and sham 6-Hz (high-frequency; excitatory) rTMS on plasticity changes induced by 1-Hz (low-frequency; inhibitory) rTMS on the hand motor cortex. Their results showed that a short bout of 6-Hz rTMS, regardless of constancy of frequency, provoked stronger and longer suppression of cortical excitability induced by 1-Hz rTMS compared to no preconditioning. This 1 Hz-preconditioned 6-Hz protocol has been tested later in a small group of stroke patients for its safety [21]. No major adverse effects were reported. However, the efficacy of such preconditioned protocol for stroke rehabilitation remained limited and therefore required further investigations.

Apart from using rTMS, different combinations of NIBS techniques, including tDCS, theta burst stimulation (TBS), and quadri-pulse stimulation (QPS), have been used to study metaplasticity of the hand motor cortex in healthy adults [22]. In all combinations, there was evidence that the state of cortical excitability preceding NIBS can be controlled within a reasonable physiological range using preconditioned stimulation.

## Promoting neuroplasticity in swallowing motor cortex through neurostimulation

In early animal studies, Sumi [23] showed that chewing and swallowing responses can be elicited by applying electrical pulses to areas that are anterolateral in the frontal lobe or rostrolateral to the postcentral area of the cortex of anaesthetized rabbits. The study found that both swallowing and chewing are bilaterally innervated within the central nervous system. Interestingly, additional stimulation from the superior laryngeal nerves facilitated cortically induced swallowing, suggesting the bi-directional synergetic effects between sensory inputs and motor control of swallowing. A later study with awaked primates demonstrated that swallowing and orofacial muscles are controlled and mediated by distinct regions of the cerebral cortex using intracortical microstimulation [24]. These regions included the lateral region of face primary motor cortex (face-MI), the lateral face primary somatosensory cortex (face-SI), the area lateral and anterior to face-MI corresponding to the cortical masticatory area (CMA), and a deep cortical area corresponding to the white matter underlying the CMA and the frontal operculum [24]. These findings along with a number of other animal-based physiologic studies of swallowing [25] showed the swallowing motor system, unlike the limb motor system, is innervated by both hemispheres and is controlled by specific areas of the cerebral cortex.

Following the development of TMS, researchers began to explore the cortical representation of human swallowing musculature in awake human subjects non-invasively [26, 27]. Aziz et al. [26] showed that both early and late electromyographic responses could be elicited in the human esophagus following single-pulse TMS and these responses were probably mediated by two different neurological pathways because of the distinct neurophysiological characteristics. The early responses might be mediated by a paucisynaptic pyramidal pathway from frontoparietal cortex to brainstem reticular formation whereas the late responses might be mediated by polysynaptic extrapyramidal pathways that were involved in swallowing control [26]. A further study by Hamdy et al. [27] showed that human swallowing musculature, including pharyngeal, esophageal, and mylohyoid muscles, are discretely and somatotopically represented in the motor and premotor cortex. Mylohyoid muscles have been reported to be represented in the lateral precentral and inferior frontal gyri, whereas pharyngeal and esophageal muscles are represented in the anterolateral precentral and middle frontal gyri and in the anterolateral precentral and superior frontal gyri respectively [27]. These representations are bilateral but asymmetrical in the two hemispheres, independent of handedness. Moreover, studies using PET and fMRI showed that swallowing recruits multiple cerebral regions, predominantly in sensorimotor areas of the brain [28, 29]. Most recently, Hashimoto et al. demonstrated for the first time the brain oscillation changes between voluntary and involuntary swallowing with electrocorticogram (ECoG) [30]. They found that the swallowing driving force switched from the cortex to brain stem at the transition from oral to pharyngeal phase of swallowing, providing evidence of the dynamics within the neural network of swallowing.

The evidence of plasticity in human swallowing motor cortex can be seen in patients who had dysphagia after stroke. Majority of the patients recover from dysphagia within weeks of stroke episode. This remarkable ability to restore swallowing function is believed to be driven by plasticity. In the study by Hamdy et al. [27], the changes in cortical pharyngeal representations of two stroke patients, one with dysphagia who fully recovered after 3 months and one without dysphagia, were examined at presentation and 3 months after stroke. They observed that at stroke presentation, the dysphagic patient had a smaller area of pharyngeal representation in the unaffected hemisphere than the non-dysphagic patient. This suggested that dysphagia may be resulted from damage to the hemisphere with larger representation (dominant hemisphere) such that the inputs from the unaffected, non-dominant hemisphere remain inadequate for functional swallowing. Moreover, the recovery of swallowing is related to an increase in the size of pharyngeal representation [27]. This study provided new evidence that plastic changes within the neural network for swallowing occur in response to a damage and play a critical role in functional recovery. Such observations were later corroborated by a further longitudinal TMS study [31]. The cortical representation of the pharynx in 28 unilateral hemispheric stroke patients was investigated at 1 week, and at 1 and 3 months after stroke. Among patients who had dysphagia initially, 75% recovered within first month after stroke. This recovery was associated with an increase in the pharyngeal representation of the unaffected hemisphere.

Taken together, these findings showed that swallowing musculature are asymmetrically represented in the two hemispheres and damage to the dominant hemisphere is likely to result in dysphagia. Importantly, recovery of swallowing function following unilateral stroke is driven by compensatory reorganization of the intact hemisphere. Facilitating such reorganization could therefore be a viable treatment goal for neurostimulation.

### Peripheral neurostimulation

The findings on the course of recovery in stroke patients serve as a model for the development of dysphagia treatments that drive plasticity changes in dysphagic patients. This can be achieved via two forms of neurostimulation: peripheral or central neurostimulation. Peripheral neurostimulation targets the afferent pathways for swallowing whereas central neurostimulation targets the central brain control of swallowing. The swallowing neuroplastic effects of human peripheral neurostimulation were first explored by Hamdy et al. [32], who studied the changes in cortical excitability in 8 healthy adults following intraluminal pharyngeal electrical stimulation (PES). The results showed that sensory inputs from electrical stimulation of pharyngeal muscles could drive an increase in cortical excitability that lasted longer than the stimulation duration. It is likely that the sensory fibers in cranial nerves IX (glossopharyngeal) and X (vagus) were activated by PES [33]. In a further study, Fraser et al. [34] identified that there was frequency specificity to the stimulation, which may be related to the conduction time for sensory inputs from the pharynx to reach the sensorimotor cortex and the optimal time window for persistent depolarization of neuronal membrane to occur. This study also verified cortical excitation with fMRI results showing increased cortical activation in the swallowing network after PES. Moreover, it showed that in acute stroke patients with dysphagia, PES could increase pharyngeal cortical excitability of the intact hemisphere, which was associated with functional recovery of swallowing.

There are limited data on the effects of PES for patients with other neurological disease. A small randomized controlled trial found that PES could reduce penetration and aspiration in patients with multiple sclerosis [35]. Moreover, a recent multi-center open-label cohort study showed that PES improves swallowing in patients with neurogenic dysphagia associated with stroke, traumatic brain injury, mechanical ventilation, or tracheostomy [36].

Apart from electrical stimulation, sensory stimulation that involves the senses of smell [37], taste [38], and vision [39] has also been employed in an attempt to alter cortical excitability or activation of the swallowing motor cortex. Ebihara et al. [37] found that prolonged (30 days) nasal inhalation of black pepper oil could induce plasticity changes in the left insular cortex, as revealed by single-photon emission computed tomography, and improve the swallowing reflex in patients with post-stroke dysphagia. Mistry et al. [38] demonstrated that both sweet (glucose) and bitter (quinine) stimuli reduced cortical excitability of the pharyngeal motor cortex. In another study by Abdul Wahab et al. [40], the authors showed that cortical excitability could only be increased by a combination of, but not separately, olfactory and gustatory stimuli and such increase lasted for at least 90 min. A further study by this group showed that such stimulation resulted in changes in swallowing biomechanics, including increase in duration and pressure of tongue-to-palate contact during swallowing [41]. Finally, food-related disgust pictures were found to reduce cortical excitability, possibly due to a triggering of avoidant-defensive mechanisms within the human swallowing system [39]. Taken together, olfactory, gustatory, and visual stimuli can either facilitate or suppress swallowing and such changes are mediated by the central nervous system.

Moreover, thermal and chemical stimulation could also alter cortical excitability of the swallowing motor cortex. Studies have shown that cold (15 °C) thermal stimulation on the tongue could increase excitability of the pharyngeal motor cortex, providing evidence of interaction between the tongue and pharyngeal cortical areas and between the afferent and efferent neural pathways [42, 43]. Moreover, a study by Elshukri et al. [44] found that carbonated boluses could increase cortical excitability that lasted for up to 60 min following the bolus inputs. Recently, a study using high-resolution manometry found that cold, sour, and carbonated boluses could increase pharyngeal contraction during swallowing in dysphagic patients [45].

Taken together, these neurophysiological findings suggest that the sensory neural pathways of swallowing could be manipulated through peripheral neurostimulation to facilitate plasticity changes in the motor cortex, making neurostimulation methods such as these a potentially viable technique for dysphagia treatment.

### Central (cortical and cerebellar) neurostimulation

Studies have shown that central neurostimulation using NIBS, including tDCS and rTMS, can induce plastic changes in human swallowing (pharyngeal) motor cortex of healthy individuals [12, 46]. An early study by Jefferson et al. [47] demonstrated that anodal (excitatory) tDCS (1 mA for 20 min or 1.5 mA for 10 min) could induce long-lasting increases in cortical excitability in healthy participants. Similarly, rTMS was found to be able to induce LTP- and LTD-like plasticity changes in the pharyngeal motor cortex. Gow et al. [46] found that the induction of plasticity is frequency-specific, with 5-Hz rTMS eliciting the most significant and longest-lasting increase in the human pharyngeal motor cortex (up to 1 h) after stimulation. For low-frequency rTMS, Mistry et al. [12] found that 1-Hz rTMS presented at 120% pharyngeal resting motor threshold could reduce cortical excitability in pharyngeal motor cortex of the stimulated hemisphere, an effect that lasted for up to 45 min. Moreover, stimulation of the dominant, but not the non-dominant, pharyngeal hemisphere could temporarily disrupt swallowing behavior. Their findings showed that 1-Hz rTMS can be used to induce a “virtual lesion” in healthy adults, which provides a model that mimics disruption of swallowing motor pathways after brain damage. The virtual lesion approach thus allows investigation of potential treatment strategies for patients with swallowing disorders. Several studies have shown that both peripheral and central neurostimulation could be applied to reverse this virtual lesion in the pharyngeal motor cortex [11, 48–50], giving credence to their likely usefulness in patient populations.

**Fig. 2:**
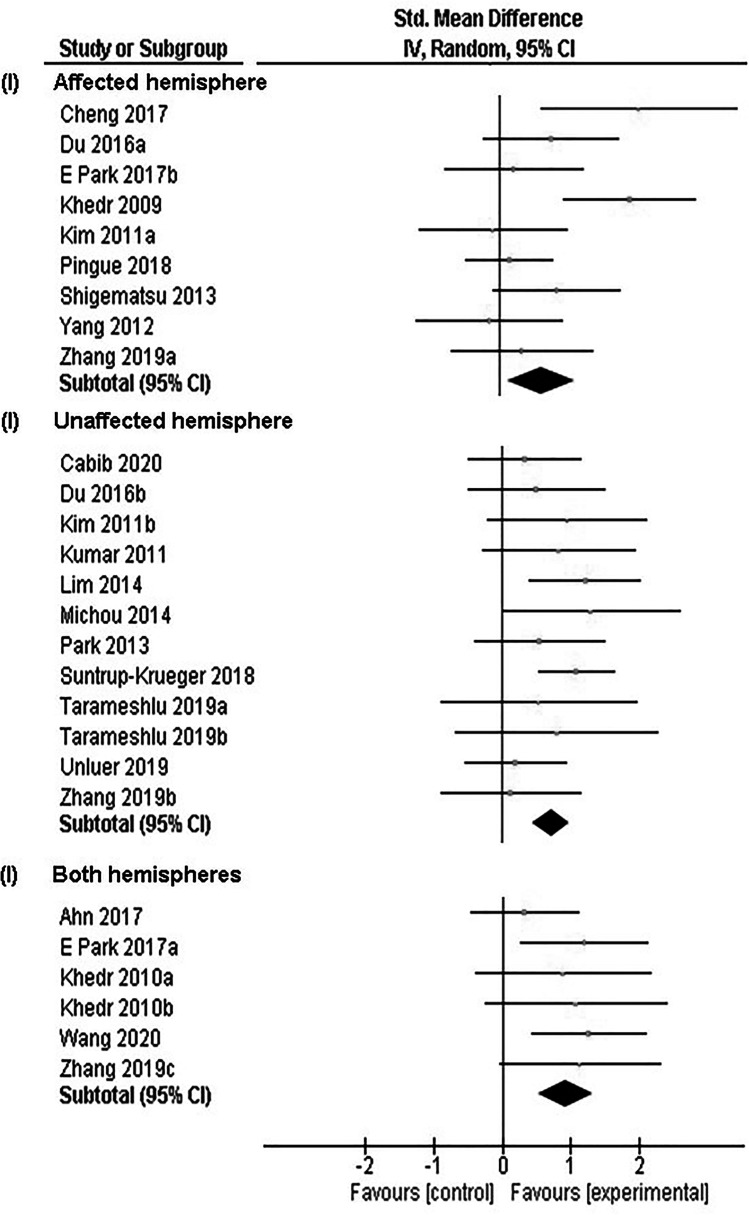
Simplified forest plot adapted from Cheng et al. [2] showing treatment effects of cortical neurostimulation based on stimulation hemisphere, including affected hemisphere, unaffected hemisphere, and both hemispheres, on swallowing functions in stroke patients. All three approaches showed beneficial effects when compared with control treatment (conventional dysphagia therapy or sham neurostimulation). Bihemispheric stimulation had the largest pooled effect size among all approaches

Over the past decade, clinical studies have demonstrated positive effects of NIBS in patients with post-stroke dysphagia [2]. Low-frequency rTMS has been used to suppress cortical excitability of the unaffected hemisphere to reduce interhemispheric inhibition [51–54], while high-frequency rTMS applied to the affected hemisphere was used to increase cortical excitability and overcome interhemispheric imbalance [52, 55]. Moreover, studies have shown that by increasing cortical excitability of the unaffected hemisphere, it is possible to improve swallowing function, presumably through facilitating reorganization of the compensatory neural network [50, 56, 57]. Some studies explored 10-Hz (high-frequency) rTMS over both hemispheres and found that bilateral rTMS improved swallowing more significantly than unilateral rTMS or sham rTMS [58–60]. Unlike rTMS, tDCS protocols are less diverse. Almost all studies in the literature employed anodal (excitatory) tDCS, over either the affected hemisphere [61–63], unaffected hemisphere [64, 65], or both hemispheres [66, 67]. A recent systematic review suggested that cortical neurostimulation is beneficial in improving swallowing in stroke patients [2]. Of note, studies have shown positive effects in both excitatory and inhibitory stimulation of the unaffected hemisphere. These seemingly contradictory outcomes may reveal the impact of stroke severity on the response to brain stimulation. It is possible that patients with severe stroke may be more responsive to stimulation that promotes reorganization of compensatory neural network (excitatory stimulation on unaffected hemisphere). Contrarily, those with less severe stroke may be more responsive to stimulation that lowers interhemispheric inhibition and allows reorganization of the residual neural networks within the affected hemisphere (inhibitory stimulation on unaffected hemisphere). Moreover, studies have shown that chronic stroke patients may have maladaptive neuroplastic changes in the affected hemisphere that hinder recovery of swallowing function [56]. Therefore, inhibitory stimulation may be useful to suppress such changes. However, these explanations remain speculative without analysis of data stratified according to stroke severity and chronicity. Importantly, bihemispheric stimulation appeared to yield better outcomes compared to unihemispheric stimulation (Fig. 2). This agrees with animal data which showed that electrical stimulation on both hemispheres elicited more frequent swallowing than the sum of swallows elicited separately from stimulation on each hemisphere [23]. Bihemispheric stimulation may have synergetic effects and result in more significant plasticity changes and functional recovery.

Similar to peripheral neurostimulation, there are limited data on the effects of central neurostimulation on dysphagic patients with other neurological diseases. A pilot study showed that 5-Hz rTMS improved swallowing and cortical activation in elderly patients with dysphagia [68]. Moreover, a prospective controlled trial found that both tDCS and intermittent theta burst stimulation (iTBS), which is a form of patterned TMS and is considered excitatory, had beneficial effects in elderly patients with dysphagia [69]. In patients with Parkinson’s disease, sequential application of 20-Hz rTMS to both hemispheres for 10 days followed by 5 booster sessions every month for 3 months reduced dysphagia severity and improved pharyngeal transit time and hyoid elevation [70]. The rTMS protocol used in this study was different from other dysphagia studies in several ways. Firstly, the stimulation frequency was higher (20 Hz) than other high-frequency studies which usually used 5-Hz or 10-Hz rTMS. Secondly, there were booster sessions, which have not been explored in other trials. Finally, rTMS was applied over the hand motor cortex instead of swallowing motor cortex. The authors argued that the improvements may be due to spreading of excitation to the adjacent esophageal motor cortex, or enhancement of functional connectivity of swallowing network by stimulation of the primary motor cortex [70]. Apart from non-invasive brain stimulation, studies have also investigated the effects of deep brain stimulation (DBS) on swallowing function in patients with Parkinson’s disease. However, the results were conflicting and there was not enough evidence to show the effectiveness of DBS in improving swallowing [71]. Finally, a small randomized controlled trial demonstrated that tDCS reduced dysphagia severity and increased cortical excitability in patients with multiple sclerosis [72].

Apart from cortical stimulation, some studies have also explored the effects of stimulation on the cerebellum, which has been shown involved in swallowing [73–75]. Physiological studies with healthy volunteers have shown that cerebellar rTMS could modulate excitability of the pharyngeal motor cortex [76] and 10-Hz rTMS appeared to be optimal for increasing such cortical excitability [77]. A further study found that 10-Hz rTMS could reverse the neurological and behavioral disruptions caused by a “virtual lesion” of the pharyngeal motor cortex [78]. Similar to cortical stimulation, bilateral cerebellar stimulation showed stronger facilitatory effects compared to unilateral stimulation [79]. Interestingly, the effects of cerebellar neurostimulation appear to be site-specific. Studies have found that rTMS or tDCS applied over the midline of cerebellum result in suppressive effects on pharyngeal cortical excitability and swallowing behavior or skill learning [80, 81]. While cerebellar neurostimulation may appear to have therapeutic potential for dysphagia, most of the data come from healthy volunteer studies. A single-patient case-controlled study found that cerebellar rTMS improved swallowing safety and increased cortical excitability in a patient with right posterior fossa infarction [82]. Recently, a quasi-randomized controlled trial found that 5-Hz suprathreshold (110% mylohyoid motor threshold) rTMS applied over the cerebellum showed beneficial effects on swallowing function in patients with post-stroke dysphagia comparable to rTMS applied over affected or unaffected hemispheres [83]. Further studies are warranted to fully explore the clinical efficacy of cerebellar neurostimulation in dysphagic patients.

Despite these positive findings, substantial variability in the responses toward NIBS treatments has been reported [84]. This prevents a generalized application and translation into clinical practice. The possible factors for such variability include genetic predisposition, brain configuration, and brain state prior to stimulation [3]. Given the intrinsic metaplastic mechanisms of the brain that regulates its response to plasticity-inducing stimulation, it is reasonable to hypothesize that preconditioning of the motor cortex prior to neurostimulation could minimize the response variability and subsequently enhance treatment outcomes.

## Metaplasticity in human swallowing motor cortex: preliminary evidence

Very few studies have explored metaplasticity mechanism in the human swallowing motor cortex. An early study by Michou et al. [85] found that “non-responders” to paired associative stimulation (PAS), which is a form of NIBS, can be switched to “responders” by delivering an additional dose of PAS within an hour. This result could not be explained by the BCM model of metaplasticity, in which double exposure of excitatory stimulation should have resulted in an overall neutral or inhibitory response. The authors suggested that the capacity or threshold for triggering a metaplasticity mechanism in the swallowing system has not been well-studied and that the protocols employed may not be the most appropriate for inducing metaplasticity. Moreover, the inter-PAS interval may play a role in affecting the results. It is also possible that the non-responders had opposite response to excitatory stimulation (inhibition) such that the first stimulation had artificially preconditioned the motor cortex into an inhibited state and lowered its threshold for LTP. When the motor cortex received the second dose of stimulation, LTP (expected response) was induced due to metaplasticity.

Recently, Cheng et al. [86] explored the induction of metaplasticity in the human swallowing motor cortex. They compared different preconditioned rTMS protocols (excitatory: 5-Hz rTMS preconditioned with 1-Hz rTMS; and inhibitory: 1-Hz rTMS preconditioned with 5-Hz rTMS) with varying inter-stimulation interval in 28 healthy volunteers. Their study showed that the pharyngeal motor cortex exhibited bi-directional metaplasticity properties in which inhibitory and excitatory effects of rTMS on cortical excitability and swallowing behavior could be enhanced by preconditioned rTMS. Moreover, there appeared to be a critical period within which metaplasticity could be induced. Specifically, the optimal inter-stimulation interval was 30 min for the 5-Hz rTMS preconditioned with 1-Hz rTMS protocol and 90 min for the 1-Hz rTMS preconditioned with 5-Hz rTMS protocol. This suggests that metaplastic changes could only take place when the after-effects of the preconditioning rTMS had built up to an adequate level. This study is an important milestone for dysphagia rehabilitation because it provides evidence that metaplasticity in the human swallowing system is functionally relevant and can be harnessed therapeutically. These findings encourage further investigations as to whether such homeostatic metaplasticity exists in patients with neurogenic dysphagia and how treatment outcomes can be improved through preconditioning of the swallowing motor cortex.

## Future research direction: cross-modal preconditioning

Apart from cortical stimulation, the motor cortex may potentially be preconditioned with other forms of plasticity-inducing stimulation, for example, peripheral (electrical, sensory, chemical, or thermal) neurostimulation. Moreover, it is not known whether preconditioning the motor cortex before traditional dysphagia therapy (that might involve behavioral exercises) would result in better treatment outcomes. Although little is known in the field of dysphagia rehabilitation, we could hypothesize the clinical usefulness of cross-modal preconditioned treatments based on the evidence from limb motor function rehabilitation trials. For example, studies have shown that plasticity induced by motor training can be enhanced by eliciting homeostatic metaplasticity [19]. Prior to motor training, neural activity could be suppressed by cortical stimulation to lower the threshold for LTP-like plasticity. Indeed, Ziemann et al. [87] found that better outcomes on a thumb abduction movement training could be achieved when participants have received LTD-like plasticity-inducing brain stimulation prior to the training than without preconditioning. Recently, a study with Parkinson’s disease patients also demonstrated that the outcomes of motor learning were more significant and sustainable after preconditioning with rTMS compared to without preconditioning [88].

Given these encouraging results from limb motor function studies, it seems reasonable to hypothesize that similar principles may be applicable to swallowing exercise training. Behavioral rehabilitative exercises, including strength training, skill-based training, and combinatory approaches, have shown positive physiological effects [89, 90]. Of relevance, Malandraki et al. [91] reported that an 8-week lingual resistance exercise regime could increase cortical activation during swallowing and lingual pressure in a chronic stroke patient. At the initial phase of training, the patient displayed perilesional activity in the affected hemisphere. However, after 8 weeks of training, there was an increase in activation in primary motor cortex, primary sensory cortex, premotor area, and insula in both hemispheres and such changes were associated with increased lingual pressure. This finding suggested behavioral exercises could induce reorganization of the neural network.

Linking the above findings to the future application of metaplasticity to swallowing rehabilitation, some studies have suggested that NIBS (tDCS and rTMS) combined with dysphagia therapy, which involves behavioral exercises, is superior to therapy alone in improving swallowing in stroke patients [53, 54, 92, 93]. While we cannot be certain about the underlying mechanism that drove this superiority, and whether cortical neurostimulation acted as prelude to inducing metaplasticity and in preparing the swallowing system for other therapeutic inputs, these findings demonstrated that when cortical neurostimulation is combined with behavioral therapy, the outcomes could be synergised. This sheds lights on the therapeutic potential of cross-modal treatments. Future studies may investigate the underlying mechanisms by manipulating other parameters of the protocol, for example, the interval between the two forms of treatments, and whether excitatory and inhibitory stimulation preceding behavioral therapy would result in opposite outcomes. Moreover, given the neurophysiological findings of peripheral (sensory, electrical, chemical, and thermal) neurostimulation, it would be interesting to investigate whether these methods could also be used as preconditioning inputs before cortical neurostimulation, or vice versa, to induce metaplasticity and enhance overall treatment outcomes.

## Conclusions

Rehabilitation of neurogenic dysphagia is thought to be mainly driven by neuroplasticity, which involves modulation of synaptic strength. Studies have shown that both peripheral and central (cortical and cerebellar) neurostimulation treatments can promote such neuroplasticity in patients with neurogenic dysphagia. Peripheral neurostimulation is mainly used to increase sensory input to the central nervous system whereas central brain stimulation is used to directly induce plasticity changes in the cortex or cerebellum. However, the response to these techniques is highly variable, possibly due to genetic variability across individuals or variations in levels of brain activation prior to stimulation. Therefore, it is important to develop robust strategies to improve treatment outcomes. Recently, the concept of metaplasticity, which is the higher order brain function that regulates responses to plasticity changes, has been explored in the human swallowing motor system. There is preliminary evidence that the outcomes of brain stimulation can be enhanced through metaplasticity induced by preconditioning of the cortex. However, it is not yet known whether metaplasticity can be induced in patients with neurogenic dysphagia with similar preconditioned protocols. Nonetheless, the concept of metaplasticity allows further exploration on the effects of preconditioning, potentially through combinations of different modalities (peripheral or central), on the human swallowing motor system and its therapeutic values in promoting recovery of neurogenic dysphagia.

## Data Availability

Not applicable.
